# Geschlechtsspezifische Unterschiede im chirurgischen Selbstvertrauen: Ergebnisse des Endo-Workshops der German Society of Residents in Urology 2022

**DOI:** 10.1007/s00120-024-02429-w

**Published:** 2024-08-27

**Authors:** Carolin Siech, Luis A. Kluth, Mareen Konopka, Maximilian Reimann, Henning Plage, Isabel Lichy, Benedikt Gerdes, Jan Kasperek, Clara Humke, Phillip Marks, Margit Fisch, Pierre I. Karakiewicz, Felix K. H. Chun, Timm Schäfer, Christian P. Meyer, Julia C. Kaulfuss

**Affiliations:** 1 Goethe-Universität Frankfurt, Universitätsklinikum, Klinik für Urologie, Theodor-Stern-Kai 7, 60590 Frankfurt am Main, Deutschland; 2 Cancer Prognostics and Health Outcomes Unit, Division of Urology, University of Montréal Health Center, Montréal, Québec, Kanada; 3 Klinik für Urologie, Charité-Universitätsmedizin Berlin, Berlin, Deutschland; 4 Klinik für Urologie, Universitätsklinikum Hamburg-Eppendorf, Hamburg, Deutschland; 5 Universitätsklinik für Urologie, Campus OWL, Klinikum Herford, Ruhr-Universität Bochum, Herford, Deutschland

**Keywords:** Simulationstraining, Operative Ausbildung, Urologische Weiterbildung, Endourologie, GeSRU, Simulation-based training, Surgical education, Residency, Endourology, GeSRU

## Abstract

**Hintergrund:**

Simulationstrainings gewinnen in der Ausbildung von Ärzt:innen in Weiterbildung (ÄiW) an Bedeutung.

**Ziel der Arbeit:**

Mit dieser prospektiven Arbeit wurde der Einfluss des Endo-Workshops der German Society of Residents in Urology e. V. (GeSRU) auf das chirurgische Selbstvertrauen evaluiert.

**Material und Methoden:**

Der GeSRU Endo-Workshop 2022 umfasste ein Simulationstraining zur Steinsanierung mittels Ureterorenoskopie (URS) und zur transurethralen Resektion der Blase (TURB). Mittels Online-Fragebogen wurde das chirurgische Selbstvertrauen vor und nach dem Workshop erhoben. Das operative Assessment erfolgte mittels Global Rating Scale (GRS).

**Ergebnisse:**

Von 40 Teilnehmenden waren 25 (62,5 %) männlich und 15 (37,5 %) weiblich. In der URS-Aufgabe erreichten männliche vs. weibliche Teilnehmende durchschnittlich 26,6 vs. 26,1/35 Punkte der GRS (*p* = 0,7) und schlossen die Aufgabe in 8,1 ± 1,9 vs. 9,9 ± 0,4 min ab (*p* < 0,001). In der Durchführung der TURB erreichten männliche vs. weibliche Teilnehmende durchschnittlich 26,0 vs. 27,3/35 Punkte der GRS (*p* = 0,3) und benötigten hierfür 7,6 ± 1,9 vs. 7,7 ± 2,2 min (*p* = 0,9). Unter den Teilnehmenden mit beantworteter Basisbefragung und Evaluation (*n* = 33), hatten 16 (80 %) männliche vs. 3 (23 %) weibliche vor (*p* = 0,01) und 19 (95 %) männliche vs. 7 (54 %) weibliche Teilnehmende nach dem Workshop (*p* = 0,03) ein chirurgisches Selbstvertrauen, eine URS durchzuführen. Bezüglich der Durchführung einer TURB hatten 10 (50 %) männliche vs. 7 (54 %) weibliche vor (*p* = 0,1) und 15 (75 %) männliche vs. 10 (77 %) weibliche nach dem Workshop ein chirurgisches Selbstvertrauen (*p* = 1,0). Eine Zunahme des chirurgischen Selbstvertrauens für die Durchführung einer URS bzw. TURB hatten 9 (45 %) bzw. 10 (50 %) männliche und 9 (69 %) bzw. 8 (62 %) weibliche Teilnehmende.

**Schlussfolgerung:**

Simulationsbasiertes Training steigert das chirurgische Selbstvertrauen. Bei vergleichbaren operativen Ergebnissen gehen weibliche ÄiW mit einem geringeren chirurgischen Selbstvertrauen an eine URS heran.

**Graphic abstract:**

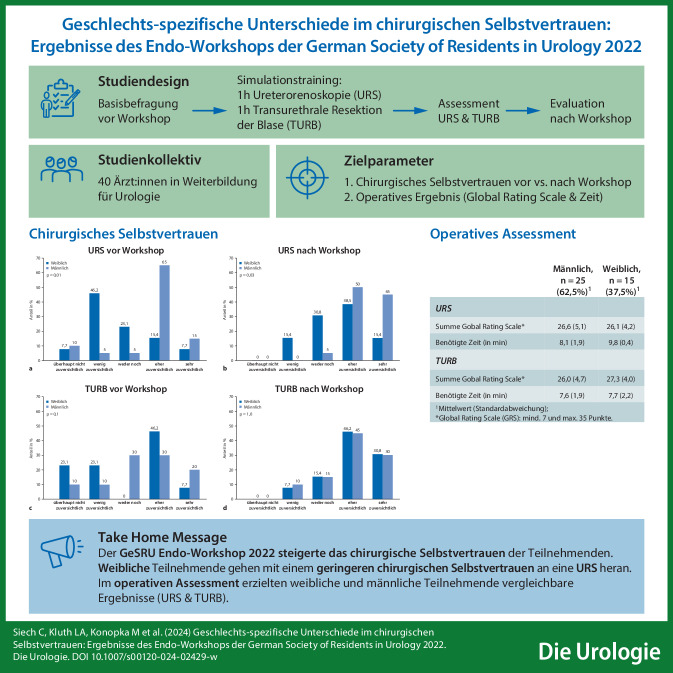

## Abstract (en)

**Background:**

Simulation-based training is gaining importance in urologic residents training.

**Objectives:**

This prospective study evaluated the influence of the Endo Workshop of the German Society of Residents in Urology e. V. (GeSRU) on surgical confidence.

**Materials and methods:**

GeSRU Endo Workshop 2022 included 1 h simulation-based training sessions on stone removal using ureteroscopy (URS) and transurethral resection of the bladder (TURB). Using an online questionnaire, surgical confidence was assessed before and after the workshop. Surgical assessment relied on the global rating scale (GRS).

**Results:**

Overall, 40 residents participated: 25 (62.5%) men and 15 (37.5%) women. In URS assessment, men vs. women achieved an average of 26.6 vs. 26.1/35 points on the GRS (*p* = 0.7) and completed the task in 8.1 ± 1.9 vs. 9.9 ± 0.4 min (*p* < 0.001). In TURB assessment, men vs. women achieved an average of 26.0 vs. 27.3/35 points on the GRS (*p* = 0.3) and required 7.6 ± 1.9 vs. 7.7 ± 2.2 min (*p* = 0.9), respectively. Among participants who answered the baseline survey and the evaluation (*n* = 33), 16 (80%) men vs. 3 (23%) women had surgical confidence to perform URS before (*p* = 0.01), and 19 (95%) men vs. 7 (54%) women after the workshop (*p* = 0.03). Regarding the performance of TURB, 10 (50%) men vs. 7 (54%) women reported surgical confidence before (*p* = 0.1), and 15 (75%) men vs. 10 (77%) women after the workshop (*p* = 1.0). An increase in surgical confidence to perform URS and TURB was reported by 9 (45%) and 10 (50%) men and 9 (69%) and 8 (62%) women, respectively.

**Conclusions:**

Endourologic simulation-based training increases surgical confidence of both female and male residents. Despite comparable surgical outcomes, women approach URS with lower surgical confidence compared to their male counterparts.

## Einleitung

Das strukturierte Erlernen operativer Fähigkeiten ist elementarer Bestandteil der urologischen Weiterbildung [3]. Es kommt jedoch im klinischen Alltag oft zu kurz [1, 4, 13, 14]. Einerseits reicht das zumeist praktizierte Paradigma „see one, do one, teach one“ nicht mehr aus, um chirurgische Kernkompetenzen sicher zu vermitteln und hierbei auch ethischen und ökonomischen Anforderungen gerecht zu werden [6, 19, 24]. Andererseits sind Methoden wie Videotraining, Simulationstraining und Mental Training als Trainingsmöglichkeiten mittlerweile zwar validiert, finden jedoch wenig Anwendung in den Ausbildungscurricula [2, 9, 11, 21, 23, 24]. Darüber hinaus nimmt der Anteil an Kolleginnen in der Urologie stetig zu [5, 28]. Eine gleiche Förderung aller Geschlechter im Bereich der operativen Ausbildung erfordert auch das Erkennen von möglichen Geschlechterunterschieden beim Erlernen neuer Techniken, um eine individuellere Förderung zu ermöglichen [18, 20, 26].

Eine außerklinisch organisierte Möglichkeit zum Auf- und Ausbau operativer Kompetenzen für Ärzt:innen in Weiterbildung (ÄiW) ist der Endo-Workshop der German Society of Residents in Urology e. V. (GeSRU; [16]). Dieser wurde im Jahr 2022 im Rahmen des 74. Jahreskongresses der Deutschen Gesellschaft für Urologie e. V. (DGU) durchgeführt. Neben einer möglichen Ergänzung zur klinischen operativen Ausbildung der Teilnehmenden bietet der Workshop zudem die Möglichkeit, im Rahmen einer begleitenden prospektiven Studie, Fragestellungen zur endourologischen Ausbildung in Deutschland, wie beispielsweise zum chirurgischen Selbstvertrauen von ÄiW zu erheben. Spezifisch postulierten wir, dass der GeSRU Endo-Workshop 2022 zu einer Zunahme des chirurgischen Selbstvertrauens führt. Darüber hinaus postulierten wir, dass männliche und weibliche Teilnehmende vergleichbare operative Ergebnisse erzielen und keine relevanten Unterschiede im chirurgischen Selbstvertrauen sowohl vor als auch nach dem Workshop zwischen den Geschlechtern bestehen.

## Material und Methoden

### Ablauf des Simulationstrainings und Studiendesign

Der GeSRU Endo-Workshop 2022 wurde im Rahmen des 74. Jahreskongresses der DGU im Olympus-Trainingscenter Hamburg durchgeführt. Es erfolgten drei konsekutive Durchläufe des jeweils dreistündigen Workshops. Dieser umfasste neben einer theoretischen Einführung jeweils ein einstündiges Simulationstraining zur Steinsanierung mittels Ureterorenoskopie (URS) und zur transurethralen Resektion der Blase (TURB). An jeweils 4 Trainingseinheiten zur URS und 4 Trainingseinheiten zur TURB mit realitätsnahen Präparaten hatten die Teilnehmenden die Gelegenheit, sich zunächst mit dem Instrumentarium vertraut zu machen und die entsprechenden Eingriffe (URS bzw. TURB) am Modell zu üben (Hands-on-Training). Die Anleitung und Überwachung der Teilnehmenden erfolgte durch erfahrene Tutor:innen (Ärzt:innen im 5. Weiterbildungsjahr sowie Fachärzt:innen). Zielgruppe des Workshops waren ÄiW für Urologie.

Die prospektive wissenschaftliche Begleitung des Workshops umfasste 3 Teile: 1) Basisbefragung (vor Workshop), 2) praktisches Assessment (während Workshop) und 3) Evaluation (nach Workshop).

### Basisbefragung und Evaluation

Allen Teilnehmenden wurde 2 Wochen vor dem GeSRU Endo-Workshop neben einem Online-Modul zur Einführung in die Endourologie eine Basisbefragung zugesendet, die vor Beginn des Workshops absolviert werden musste. Zudem wurden die Teilnehmenden dazu aufgefordert, nach dem Workshop eine Evaluation auszufüllen. Die online-basierten Befragungen umfassten insgesamt 33 bzw. 30 Fragen zu Basisdaten, Berufszufriedenheit, Weiterbildung und operativen Ausbildung sowie zum chirurgischen Selbstvertrauen. Zudem wurde die Kurzfassung des validierten Fragebogens zum Modell der beruflichen Gratifikationskrisen nach Siegrist verwendet (2 Fragen mit jeweils 8 Unterfragen, Verausgabungs-Belohnungs-(ER) Ungleichgewicht-Fragebogen mit 4‑Punkte-Likert-Skala). Alle Befragungen enthielten jeweils 5 zusätzliche Fragen zur Erstellung eines persönlichen Pseudonyms der Teilnehmenden, welches zur Zusammenführung der erhobenen Datensätze diente.

### Praktisches Assessment

Im Anschluss an das praktische Simulationstraining wurde ein praktisches Assessment an der jeweiligen Trainingseinheit (URS und TURB) durchgeführt. Zur objektiven Bewertung des operativen Ergebnisses wurden als validierte Messinstrumente die Global Rating Scale (GRS) sowie die Zeitmessung angewendet. Der zeitliche Rahmen wurde pro Teilnehmenden auf maximal 10 min begrenzt. Die Überwachung und Bewertung der Teilnehmenden erfolgte durch erfahrene Tutorinnen und Tutoren.

### Studienvariablen

Primärer Studienendpunkt bildete das chirurgische Selbstvertrauen („surgical confidence“), eine URS bzw. eine TURB durchzuführen, das analog zu einer bereits publizierten Vorarbeit auf europäischer Ebene erhoben wurde [8]. Sekundärer Endpunkt stellte das operative Ergebnis im praktischen URS- bzw. TURB-Assessment dar [12]. Die Teilnehmenden wurden entsprechend ihres Geschlechtes (männlich vs. weiblich) stratifiziert.

### Statistik

Drei Analyseschritte wurden durchgeführt. Zunächst wurden die Basismerkmale tabellarisch erfasst. Die deskriptiven Statistiken umfassten Mittelwerte und Standardabweichungen (SD) für kontinuierlich kodierte Variablen sowie absolute und relative Häufigkeiten für kategoriale Variablen. Anschließend wurden der χ^2^-Test, der Welch’s t‑test und der Exakte-Test nach Fisher für Vergleiche zwischen zwei Gruppen zu einem bestimmten Zeitpunkt angewendet. Nachfolgend wurden mit einem Ein-Stichproben-Proportionstest mit Kontinuitätskorrektur Unterschiede im chirurgischen Vertrauen vor und nach dem Workshop in der Gesamtkohorte bewertet. Ein erfolgreicher Workshop wurde danach definiert, dass das chirurgische Selbstvertrauen der Teilnehmenden zwischen dem Beginn und dem Ende des Workshops um 30 % zunahm [25]. Schließlich wurden in geschlecht-spezifischen Subgruppenanalysen Zwei-Stichproben-Tests auf Gleichheit der Proportionen mit Kontinuitätskorrekturen durchgeführt.

Alle Tests waren zweiseitig, wobei das Signifikanzniveau auf *p* < 0,05 festgelegt wurde. Für die statistischen Berechnungen und die Erstellung der Grafiken wurde die Softwareumgebung R verwendet (R Version 4.2.2; R Foundation for Statistical Computing, Wien, Österreich; [22]).

## Ergebnisse

### Studienkohorte

Von den insgesamt 46 Teilnehmenden des GeSRU-Endo-Workshops 2022, hatten 40 Teilnehmende die Basisevaluation beantwortet, sodass diese in die Studienkohorte eingeschlossen werden konnten. Von diesen waren 25 (62,5 %) männlich und 15 (37,5 %) weiblich. Die Basischarakteristika der Teilnehmenden sind Tab. 1 zu entnehmen. Die durchschnittliche Anzahl der vor dem Workshop bereits selbstständig durchgeführten URS (22 vs. 18), TURB (15 vs. 22) und transurethralen Resektion der Prostata (TURP: 4 vs. 5) war bei männlichen und weiblichen Teilnehmenden vergleichbar. Vorerfahrungen mit Simulatoren hatten 10 (40 %) der männlichen und 4 (27 %) der weiblichen Teilnehmenden.

**Tab. 1:** Basischarakteristika von 40 der 46 Teilnehmenden des GeSRU-Endo-Workshops 2022 (German Society of Residents in Urology e.V.) stratifiziert nach dem Geschlecht der Teilnehmenden (männlich vs. weiblich). *TURB* Transurethrale Resektion der Blase, *TURP* transurethrale Resektion der Prostata, *URS* Ureterorenoskopie

Basischarakteristika	Insgesamt *n* = 40	Männlich *n* = 25 (62,5 %)	Weiblich *n* = 15 (37,5 %)
**Weiterbildungsjahr** (*n* [%])			
1. Weiterbildungsjahr	2 (5)	2 (8)	0 (0)
2. Weiterbildungsjahr	5 (13)	5 (20)	0 (0)
3. Weiterbildungsjahr	15 (38)	10 (40)	5 (33)
4. Weiterbildungsjahr	10 (25)	4 (16)	6 (40)
≥ 5. Weiterbildungsjahr	8 (20)	4 (16)	4 (27)
**Arbeitgeber** (*n* [%])			
Universitätsklinikum	10 (25)	4 (16)	6 (40)
Klinik der Maximalversorgung	9 (23)	4 (16)	5 (33)
Klinik der Grund‑, Regel-, und Schwerpunktversorgung	19 (48)	16 (64)	3 (20)
Sonstiges	2 (5)	1 (4)	1 (7)
**Arbeitszeitmodell: **Vollzeit (*n* [%])	37 (93)	22 (88)	15 (100)
**Weiterbildungscurriculum** (*n* [%])	16 (40)	11 (44)	5 (33)
**Weiterbildungsgespräche** (*n* [%])			
Keine	8 (20)	5 (20)	3 (20)
Regelmäßig	15 (38)	9 (36)	6 (40)
Unregelmäßig	17 (43)	11 (44)	6 (40)
**Durchschnittliche Anzahl an** **Operationen pro Woche** (*n* [%])			
< 1	5 (13)	3 (12)	2 (13)
1–3	28 (70)	16 (64)	12 (80)
4–6	5 (13)	4 (16)	1 (7)
≥ 7	2 (4)	2 (8)	0 (0)
**Bisherige Anzahl an URS** (Mittelwert [Standardabweichung])	20 (26)	22 (27)	18 (25)
**Bisherige Anzahl an TURB** (Mittelwert [Standardabweichung])	18 (19)	15 (16)	22 (25)
**Bisherige Anzahl an TURP** (Mittelwert [Standardabweichung])	4 (6)	4 (7)	5 (5)
**Erfahrung mit endourologischen Simulatoren **(*n* [%])	14 (36)	10 (40)	4 (27)

### Praktisches Assessment

Im URS-Assessment erzielten die Teilnehmenden durchschnittlich 26,5 ± 4,7 Punkte von maximal 35 Punkten (Summe GRS) und benötigten durchschnittlich 8,8 ± 1,8 min (Tab. 2). Männliche im Vergleich zu weiblichen Teilnehmenden erreichten vergleichbare operative Ergebnisse während des Workshops (Summe GRS 26,6 ± 5,1 vs. 26,1 ± 4,2 Punkte; *p* = 0,7), benötigten jedoch weniger Zeit zur Durchführung des URS-Assessments (8,1 ± 1,9 vs. 9,9 ± 0,4 min; *p* < 0,001).

**Tab. 2:** Ergebnisse des Assessments der Teilnehmenden des GeSRU-Endo-Workshops 2022 (German Society of Residents in Urology e.V.) stratifiziert nach dem Geschlecht der Teilnehmenden (männlich vs. weiblich)

Charakteristika	Insgesamt *n* = 40^a^	Männlich *n* = 25 (62,5 %)^a^	Weiblich *n* = 15 (37,5 %)^a^	*p*-Wert^b^
*URS*				
**Summe Gobal Rating Scale**^c^	26,5 (4,7)	26,6 (5,1)	26,1 (4,2)	0,7
**Benötigte Zeit (min)**	8,8 (1,8)	8,1 (1,9)	9,8 (0,4)	**<0,001**
*TURB*				
**Summe Gobal Rating Scale**^*c*^	26,5 (4,4)	26,0 (4,7)	27,3 (4,0)	0,3
**Benötigte Zeit (min)**	7,6 (1,7)	7,6 (1,9)	7,7 (2,2)	0,9

*URS* Ureterorenoskopie, *TURB* transurethrale Resektion der Blase, *min* Minuten

^a^Mittelwert (Standardabweichung)

^b^Welch Two Sample t‑test; Fisher’s exact test

^c^Global Rating Scale (GRS): mind. 7 und max. 35 Punkte

Im TURB-Assessment erzielten die Teilnehmenden durchschnittlich 26,5 ± 4,4 von maximal 35 Punkten (Summe GRS) und benötigten durchschnittlich 7,6 ± 1,7 min. Männliche und weibliche Teilnehmende erreichten vergleichbare Ergebnisse (Summe GRS vs. 26,0 ± 4,7 vs. 27,3 ± 4,0 Punkte; *p* = 0,3) bei vergleichbarer benötigter Zeit (7,6 ± 1,9 vs. 7,7 ± 2,2 min; *p* < 0,001).

### Chirurgisches Selbstvertrauen

Unter den Teilnehmenden, die sowohl Basisbefragung als auch Evaluation vollständig beantwortet haben (*n* = 33), hatten 19 (58 %) Teilnehmende vor dem Workshop und 26 (79 %) nach dem Workshop ein chirurgisches Selbstvertrauen, eine URS durchzuführen. Das chirurgische Selbstvertrauen nahm bei 18 (55 %) Teilnehmenden zu und bei keinem der Teilnehmenden ab. Für die Durchführung einer TURB hatten 17 (52 %) Teilnehmende vor und 25 (76 %) nach dem Workshop ein chirurgisches Selbstvertrauen. Das chirurgische Selbstvertrauen nahm bei 18 (55 %) Teilnehmenden zu und bei 2 (6 %) Teilnehmenden ab.

**Abb. 1:**
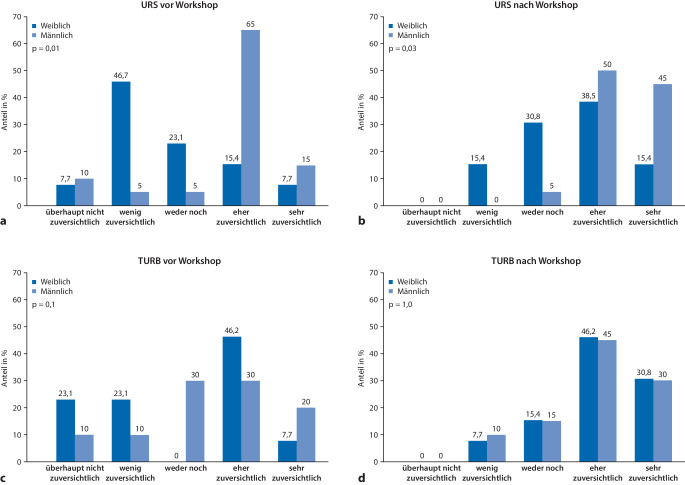
Chirurgisches Selbstvertrauen der Teilnehmenden des GeSRU-Endo-Workshops 2022 (German Society of Residents in Urology e.V.) stratifiziert nach dem Geschlecht der Teilnehmenden (männlich *n* = 20 vs. weiblich *n* = 13): **a** eine Ureterorenoskopie (URS) durchzuführen vor dem Workshop, **b** eine URS durchzuführen nach dem Workshop, **c** eine transurethrale Resektion der Blase (TURB) durchzuführen vor dem Workshop, **d** eine TURB durchzuführen nach dem Workshop (*n* = 33). *TURB* transurethrale Resektion der Blase, *URS* Ureterorenoskopie

In geschlechtsspezifischen Analysen hatten 16 (80 %) männliche vs. 3 (23 %) weibliche Teilnehmende vor dem Workshop (*p* = 0,01) und 19 (95 %) vs. 7 (54 %) nach dem Workshop (*p* = 0,03) ein chirurgisches Selbstvertrauen, eine URS durchzuführen (Abb. 1a, b). Eine Zunahme des chirurgischen Selbstvertrauens, eine URS durchzuführen, hatten 9 (45 %) männliche und 9 (69 %) weibliche Teilnehmende. Ein chirurgisches Selbstvertrauen, eine TURB durchzuführen, hatten 10 (50 %) männliche vs. 7 (54 %) weibliche Teilnehmende vor dem Workshop (*p* = 0,1) und 15 (75 %) vs. 10 (77 %) nach dem Workshop (*p* = 1,0; Abb. 1c, d). Eine Zunahme des chirurgischen Selbstvertrauens für die Durchführung einer TURB hatten 10 (50 %) männliche und 8 (62 %) weibliche Teilnehmende.

## Diskussion

In Rahmen des GeSRU-Endo-Workshops 2022 untersuchten wir prospektiv den Einfluss von simulationsbasiertem endourologischen Training (URS und TURB) auf das subjektive chirurgische Selbstvertrauen der Teilnehmenden sowie mögliche geschlechtsspezifische Unterschiede. Dabei haben wir wichtige Erkenntnisse gewonnen.

Erstens waren von den insgesamt 40 Teilnehmenden, die in die Studienkohorte eingeschlossen werden konnten, 25 (62,5 %) männlich und 15 (37,5 %) weiblich. Somit nahmen am Workshop insgesamt mehr männliche als weibliche ÄiW teil. Folglich waren männliche Teilnehmende in der Studienkohorte im Vergleich zur allgemeinen Verteilung von männlichen vs. weiblichen ÄiW für Urologie in Deutschland überrepräsentiert [1, 4]. Dies ist möglicherweise durch die Kopplung des Endo-Workshops 2022 an den Jahreskongress der DGU 2022 bedingt, da männliche Kollegen häufiger wissenschaftlich aktiv und demzufolge Kongressteilnehmende sind [1, 10, 29].

Zweitens unterschieden sich männliche vs. weibliche Teilnehmende nicht klinisch relevant in ihrer endourologischen Vorerfahrung, hatten jedoch häufiger Vorerfahrung mit endourologischen Simulatoren (40 vs. 27 %). Diese Beobachtung ist in Übereinstimmung mit den zuvor beschriebenen Unterschieden in der Geschlechterverteilung der Teilnehmenden des GeSRU-Endo-Workshop 2022. Darüber hinaus wurden auch für andere urologische Simulationstrainings eine überdurchschnittlich häufige Teilnahme von männlichen Kollegen beobachtet [7, 15]. Idealerweise sollten derartige geschlechtsspezifische Unterschiede an der Beteiligung an Simulationstrainings nicht existieren. Um diese Unterschiede anzugehen, sollten weibliche ÄiW von ihren Vorgesetzen und Kollegen zu einer Teilnahme an Simulationstrainings ermutigt und motiviert werden.

Drittens konnten wir in Bezug auf das chirurgische Selbstvertrauen feststellen, dass dieses im Vergleich vor vs. nach dem Endo-Workshop für beide endourologische Eingriffe (URS 58 vs. 79 % und TURB 52 vs. 76 %) in der gesamten Studienkohorte zunahm. Zudem wurde bei beiden Geschlechtern sowohl bei der Durchführung einer URS (45 vs. 69 %) als auch einer TURB (50 vs. 62 %) eine Zunahme des chirurgischen Selbstvertrauens beobachtet. Die aktuellen Beobachtungen validieren unsere Hypothese, dass endourologisches simulationsbasiertes Training beim Erwerb operativer Kompetenzen unterstützen kann. Ähnliche Assoziationen wurden zuvor auch zwischen laparoskopischen und robotischem Trainings und chirurgischem Selbstvertrauen auf europäischer Ebene beobachtet [8].

Viertens gehen männliche ÄiW im Vergleich zu ihren weiblichen Kolleginnen häufiger mit einem chirurgischen Selbstvertrauen an die Durchführung einer URS heran. Vor dem Workshop hatten 80 % männliche vs. 23 % weibliche Teilnehmende (*p* = 0,01) und nach dem Workshop 95 vs. 54 % (*p* = 0,03) das Selbstvertrauen, eine URS durchzuführen. Dagegen zeigten sich in Bezug auf die TURB keine signifikanten Unterscheide zwischen männlichen und weiblichen Teilnehmenden sowohl vor als auch nach dem Workshop (vor *p* = 0,1; nach *p* = 1,0). Diese Beobachtung ist von besonderer klinischer Relevanz, da sich somit weibliche ÄIW in ihren operativen Fähigkeiten häufig unterschätzen und auch von Kollegen unterschätzt werden können [17, 18, 20].

Fünftens wurden männliche und weibliche ÄiW im operativen Assessment während des Workshops mit vergleichbaren manuellen Fähigkeiten sowohl bei der Durchführung der URS (GRS 26,6 vs. 26,1 von maximal 35 Punkten) als auch der TURB (GRS 26,0 vs. 27,3 Punkten) bewertet. Es zeigten sich somit keine signifikanten geschlechterspezifischen Unterschiede in den erreichten GRS-Punktzahlen (URS *p* = 0,7; TURB *p* = 0,3). Männliche ÄiW benötigten jedoch signifikant weniger Zeit für die Durchführung der URS im Vergleich zu ihren Kolleginnen (8,1 vs. 9,8 min; *p* < 0,001). Dagegen zeigten sich keine signifikanten Unterschiede in Bezug auf die Prozedurzeiten der TURB. Diese Beobachtungen sind im Einklang mit Sun et al., der ebenfalls beobachtete, dass männliche Novizen die flexible URS schneller durchführten [27]. Nichtsdestotrotz waren die Unterschiede gering und wurden durch die Arbeitsgruppe als nicht klinisch relevant erachtet [27].

Zusammengefasst konnte in der vorliegenden Studie, die begleitend zum GeSRU-Endo-Workshop 2022 durchgeführt wurde, gezeigt werden, dass der Workshop zu einer Zunahme des chirurgischen Selbstvertrauens für die Durchführung endourologischer Eingriffe (URS und TURB) beiträgt. Dabei gehen jedoch weibliche ÄiW im Vergleich zu ihren männlichen Kollegen trotz vergleichbarer operativer Ergebnisse (GRS) seltener mit einem chirurgischen Selbstvertrauen an die Durchführung einer URS, nicht aber einer TURB heran.

Trotz ihrer Stärken ist die vorliegende prospektive Studie nicht frei von Limitationen. Zuallererst ist die begrenzte Anzahl an Studienteilnehmenden anzugeben. Diese ist u. a. dem hohen organisatorischen, personellen und finanziellen Aufwand geschuldet, der mit der Durchführung eines Simulationstrainings einhergeht. Um eine enge Betreuung von Teilnehmenden mit Tutorinnen und Tutoren zu ermöglichen, ist die Anzahl der Teilnehmenden bei Hands-on-Workshops leider zu begrenzen. Diese Limitation gilt auch für andere prospektive Studien, in denen Simulationstraining untersucht wurde [2, 7, 12, 15, 27]. Zudem beantworteten nicht alle Workshop-Teilnehmenden alle Befragungen (Basisbefragung und Evaluation), sodass lediglich 40 der 46 Teilnehmenden, in die Studienkohorte eingeschlossen werden konnten. Dies kann möglicherweise zu einer Selektionsverzerrung („selection bias“) geführt haben. Ebenso ist anzumerken, dass aufgrund des begrenzten zeitlichen Umfangs des Endo-Workshops, eine Zeitobergrenze von 10 min pro durchgeführte Übung im Assessment festgesetzt werden musste. Dies hat insbesondere bei der URS-Übung dazu geführt, dass nicht alle Teilnehmenden die vorgegebene Aufgabenstellung vollständig erfüllen konnten und somit die geschlechtsspezifischen Unterschiede der Zeitenerfassung der Prozeduren unterschätzt werden konnten.

## Schlussfolgerung

Endourologisches simulationsbasiertes Training steigert sowohl bei weiblichen als auch männlichen ÄiW das chirurgische Selbstvertrauen. Bei vergleichbaren operativen Ergebnissen gehen weibliche ÄiW im Vergleich zu ihren männlichen Kollegen mit einem geringeren chirurgischen Selbstvertrauen an die Durchführung einer URS heran.

## Fazit für die Praxis

- Der Endo-Workshop 2022 der German Society of Residents in Urology e.V. (GeSRU) steigerte das chirurgische Selbstvertrauen der Teilnehmenden.
- Im operativen Assessment erzielten weibliche und männliche Teilnehmende vergleichbare Ergebnisse in der Durchführung sowohl einer Ureterorenoskopie (URS) als auch einer transurethralen Resektion der Blase (TURB).
- Weibliche Teilnehmende gehen jedoch mit einem geringeren chirurgischen Selbstvertrauen an die Durchführung einer URS heran.
- Um neben operativen Kompetenzen auch das chirurgische Selbstvertrauen zu stärken, sollten Ärztinnen und Ärzte in Weiterbildung zu einer Teilnahme an Simulationstrainings motiviert werden.

## Data Availability

Die Studiendaten können auf begründete Anfrage zur Verfügung gestellt werden.
